# Feature Analysis of Facial Color Information During Emotional Arousal in Japanese Older Adults Playing eSports

**DOI:** 10.3390/s25185725

**Published:** 2025-09-13

**Authors:** Ryota Kikuchi, Hikaru Shirai, Chikako Ishizawa, Kenji Suehiro, Nobuaki Takahashi, Hiroki Saito, Takuya Kobayashi, Hisami Satake, Naoko Sato, Yoichi Kageyama

**Affiliations:** 1 Graduate School of Engineering Science, Akita University, 1-1 Tegata Gakuen-machi, Akita-shi 010-8502, Akita, Japan; kikuchir@gipc.akita-u.ac.jp (R.K.); shirai@ie.akita-u.ac.jp (H.S.); ishizawa@ie.akita-u.ac.jp (C.I.); 2 Cable Networks Akita Co., Ltd., 1-3 Yabase Minami-1tyoume, Akita-shi 010-0976, Akita, Japan; suehiro@cna-catv.co.jp (K.S.); nobuaki-t@cna-catv.co.jp (N.T.); h-saito@cna-catv.co.jp (H.S.); tak-koba@cna-catv.co.jp (T.K.); 3 ALL-A Co., Ltd., 1-3 Yabase Minami-1tyoume, Akita-shi 010-0976, Akita, Japan; hisami-s@all-a.jp (H.S.); naoko.sato@all-a.jp (N.S.)

**Keywords:** aging population, cognitive function, cognitive impairment, dementia, eSports, healthy life expectancy, older adults

## Abstract

Rapid digitalization has resulted in an increase in the number of older adults playing electronic sports (eSports). Therefore, it must be investigated whether eSports have a positive effect on cognitive function in older adults. We explored the traits of facial color modification in Japanese older individuals while playing eSports by employing the facial color analysis technique proposed in this study. With the aging population, eSports have garnered interest as a means of extending healthy life expectancy. The quantitative detection of emotions obtained from eSports can function as an indicator for evaluating the degree to which individuals enjoy the games and can aid in the assessment of eSports to extend healthy life expectancy. Thus, in this study, we aimed to develop an indicator for quantitatively assessing the emotions experienced while playing eSports. The investigation revealed that information on color saturation in the cheek region exhibited a distinct relationship with the emotions generated while playing eSports. The identified characteristics can also be utilized in techniques to estimate the emotions generated during eSports activities. This can contribute to the evaluation of eSports in extending the healthy life expectancy of older adults. Furthermore, it can aid in the development of technologies that support remote communication.

## 1. Introduction

Currently, older adults account for 29.3% of the total population in Japan, the highest proportion ever recorded. Among countries and regions with populations over 100,000, Japan has the highest percentage of people aged 65 and over relative to the total population [1]. Therefore, it is essential to prolong the healthy life expectancy of older adults. One of the factors hindering this is dementia. The prevalence of dementia and mild cognitive impairment among adults aged 65 years and above in Japan is increasing [2]. Therefore, the prevention of dementia and the maintenance and improvement of cognitive function are crucial.

For example, studies have investigated the relationship between the manner in which older adults connect with society and the risk of developing dementia [3]. The results revealed that this risk is reduced in older adults who are connected to people and communities from various backgrounds.

Further, computer and video games may have a positive impact on cognitive function, and electronic sports (eSports) have attracted increasing attention in recent years. eSports is a form of competition wherein individuals or teams compete via computers or video games. eSports is becoming increasingly popular among young people. Research has been conducted on the relationship between eSports and cognitive ability in young people [4,5,6,7]. For example, [4] reported an increase in visual response time before and after playing action games. Another study [5] revealed that certain areas of the brain, particularly the prefrontal cortex, are activated after playing video games. In older adults, a wide range of game genres, such as brain training and exercise games, have been studied for their relationship with cognitive function [8,9]. For example, one study [8] revealed improvements in the cognitive status of participants who play the Wii Fit game. Furthermore, [9] revealed that strategy video games activate the frontoparietal areas in older adult participants. Additionally, game design and research on more effective game-playing conditions are being conducted with the aim of utilizing games to improve cognitive function [10,11].

With recent developments in information technology, it has become easier to communicate with distant opponents, thus enabling people to interact and have fun with others through games. Therefore, eSports provides two benefits, “playing games” and “interacting with others,” and is an effective form of sport for preventing dementia and extending healthy life expectancy. However, few studies have collected data on older adults engaging in eSports or examined their physiological information and state during gameplay [12]. With rapid digitalization in recent years, the number of older adults playing eSports has increased. Therefore, it is important to investigate whether eSports has a positive effect on cognitive function in older adults.

When investigating the effects of eSports on older adults, it is important to carefully consider what information to collect and how to collect it. In general, brain function sensing methods such as electroencephalography and functional magnetic resonance imaging (fMRI) are often used to study the effects of games on the human body [5,9,13]. However, these methods are expensive owing to the need for specialized equipment. In addition, these methods may cause stress to the participants, as the equipment must be worn on their bodies for sensing purposes. Therefore, the sensing of information about older adults while they play eSports via a method that does not burden them and can easily acquire data is necessary. To address this, we focused on emotional information.

Quantifying emotions experienced during gameplay can serve as a useful indicator for evaluating how much participants enjoy the game. Furthermore, it enables the temporal analysis of emotional changes across repeated gameplay sessions, providing insights into how participants’ mental states evolve over time. In addition, [14] reported that older adults with high positive emotion scores have a significantly lower probability of developing dementia. Consequently, these indices can be used to evaluate and improve eSports for the prevention of dementia and extension of healthy life expectancy in older adults. Therefore, this study aimed to construct a system to estimate the emotions that occur while playing eSports, generate an index to quantitatively judge the emotions obtained from eSports, and scientifically clarify the effects of eSports on the extension of healthy life expectancy and prevention of dementia.

Many studies aim to make effective use of emotional information. For example, [15] investigated the integration of emotion data into search interfaces to develop more adaptive and responsive search systems for users. In another example, [16] presented a chatbot capable of emotion recognition, which enables automated responses tailored to the emotions expressed in product reviews, thereby contributing to business support in online shopping environments. 

Similarly to our approach of utilizing emotional information for analyzing user experience, numerous studies have been conducted in the field of multimedia [17,18,19]. These studies have proposed methods to evaluate users’ quality of experience during video viewing or videoconferencing, achieving high measurement accuracy. However, to the best of our knowledge, no research has utilized emotional information to analyze the experiences of users during eSports gameplay.

Traditionally, many studies investigating the use of emotions that arise during gameplay have focused on improving the performance of players or teams [20,21,22,23,24]. In addition, some works have detected emotions during gameplay and leveraged them to propose game designs [25] or to examine differences between highly profitable and less profitable games [26]. However, these studies have generally targeted young eSports players. Therefore, research that collects and analyzes data from older adults engaged in eSports gameplay, as in our study, is needed.

Emotion estimation techniques can be broadly categorized into contact-based methods, which require the attachment of sensors to the body, and contactless methods, which estimate emotional states using information acquired without physical contact. Considering the potential stress that contact-based systems may impose on participants, this study focused on contactless emotion estimation methods. One of the most widely used approaches in this category is facial expression analysis [17,18,19,27,28,29,30]. Facial expressions are closely tied to emotional states and are considered critical sources of information in human emotional recognition processes [27,31]. However, facial expressions can be voluntarily controlled, meaning that they do not always reflect a person’s true emotional state [32,33]. For example, people may smile genuinely when they are happy or smile to mask negative emotions. This makes it challenging to estimate emotions solely based on facial expressions. Moreover, previous studies have suggested that older adults—the target population of this study—may exhibit a lower degree of emotional expression through facial expressions than younger individuals [34] and that facial age may affect the interpretation of emotional expressions [35]. Voice-based emotion estimation methods also exist [19,36,37], but similarly to facial expressions, vocal features can be easily manipulated [38]. Furthermore, voice-based approaches require the participant to vocalize during gameplay, which is not always the case. Although some eSports games involve team-based communication, others are played individually, and players in solo formats may have fewer opportunities to speak than those in team settings.

In contrast, human physiological responses are regulated by the autonomic nervous system and the endocrine system and are not directly controlled by subjective thoughts [39]. Moreover, emotional arousal is known to be closely associated with autonomic nervous system activity, which induces various physiological changes [40,41]. On the basis of this relationship, we focused on facial color information as a means to estimate emotional states through physiological responses. The autonomic nervous system regulates various physiological changes such as heart rate, respiration, sweating, and skin blood flow [42]. The regulation of skin blood flow involves changes in blood volume caused by the constriction or dilation of subcutaneous blood vessels. The autonomic nervous system also controls facial muscles, and blood flow is adjusted according to muscle activity [42]. Such changes in blood volume can lead to variations in facial temperature [42] and alterations in the color of the affected areas, such as changes in skin redness [43]. Therefore, focusing on changes in a participant’s facial color while playing eSports allows for an estimation of whether the participant is experiencing certain emotions and the types of emotions that are aroused. In this study, we propose a method for analyzing facial color and investigating the characteristics of facial color change when emotions arise in older adult participants in Japan playing eSports, and report the findings of this investigation.

The objective of this study is to develop an emotion estimation system that focuses on physiological changes—which are difficult to consciously control and measurable through noncontact means—to address the four key challenges outlined below. In addition, we aim to clarify the impact of eSports on older adults through emotion-based analysis.

1. Although the extension of healthy life expectancy among older adults is a pressing issue and eSports has gained attention as a potential solution, there is a lack of research that collects data from older adults engaging in eSports or investigates their physiological and psychological states during gameplay.
2. Contact-based data collection methods that require participants to wear devices may impose a burden on them.
3. Although emotion—which can be acquired noninvasively—has potential for evaluating the benefits of eSports in extending healthy life expectancy, specific methods for leveraging emotional data have not been thoroughly examined.
4. Noncontact indicators commonly used for emotion estimation, such as facial expressions and voice, can be consciously manipulated by individuals and do not necessarily reflect their actual emotional states.

The main contributions of this study are as follows:

1. We conducted data collection and experiments focusing on older adults engaging in eSports, a domain that has been insufficiently explored to date. As eSports rapidly gain global popularity, this paper offers insights into how this emerging cultural phenomenon may impact an aging society, thereby contributing to the advancement of this research field.
2. While brain function sensing is commonly employed to investigate the effects of games on individuals, we took a novel approach by utilizing emotional information to analyze player experience (e.g., enjoyment) and psychological states during gameplay. This study has the potential to advance research in the field that leverages emotional data for user experience analysis.
3. As a method for estimating players’ emotions, we focused on a new perspective within the wide range of noncontact emotion estimation techniques—namely, changes in facial color information. Since facial color information reflects physiological changes that cannot be voluntarily controlled, the method proposed in this paper may provide a fresh perspective for current emotion estimation technologies and contribute to the development of this field.

The remainder of this paper is organized as follows: Section 2 describes the data acquisition experiment performed to investigate facial color changes. Section 3 describes the facial color analysis method employed. Section 4 presents the results of the analysis via the method described in Section 3 along with a discussion. Finally, Section 5 presents the conclusions and future research directions.

## 2. Data Acquisition

**Figure 1:**
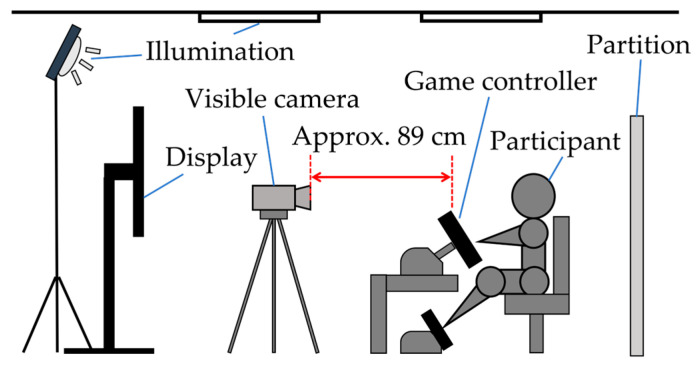
Data acquisition environment.

A data acquisition experiment was conducted to obtain data for use in this study. Figure 1 shows the experimental environment used for data acquisition. The experiment was conducted twice with 19 Japanese participants (12 male and 7 female) aged 60 years or older, each assigned an identifier from A to S. However, three participants (D, O, and S) participated in the experiment only once.

Among older adults, it was anticipated that few individuals would be familiar with playing video games on a regular basis. For those with limited gaming experience, games that require the use of dedicated controllers or keyboards may be difficult to operate and could impose stress or burden simply through the act of gameplay. Therefore, in this experiment, the participants were asked to play the racing game Gran Turismo Sport [44]. As shown in Figure 1, this game allows the use of a controller integrating a steering wheel and pedals, enabling intuitive operation. Moreover, in the local region where this study was conducted, automobiles are a familiar aspect of daily life. On the basis of these considerations, we expected that using a racing game would reduce resistance to gaming and allow participants to play with minimal stress. In fact, prior to the gameplay session, a questionnaire was administered to all the participants. The results revealed that approximately 50% of the participants had played video games at least once in the past. Among this group, only 20% reported playing video games on a regular basis. Additionally, all the participants had driving experience, and 90% of them reported that they drive regularly. The results of this questionnaire are summarized in Table 1.

**Figure 2:**
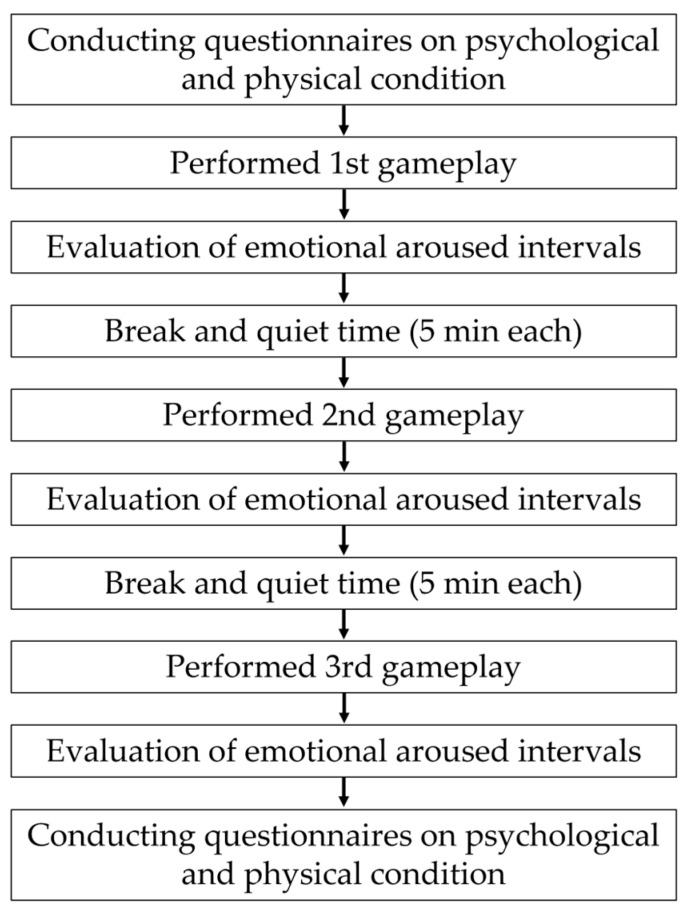
Data acquisition flow.

A flowchart of the data acquisition experiment is presented in Figure 2. First, the participants were asked to complete a questionnaire regarding their psychological and physical conditions before and after the experiment to understand their health status. The participants were then asked to play a game three times per session. The game was filmed from the front of the participant via a visible camera (Panasonic Holdings Corporation, Osaka, Japan, 4K video camera HC-VX2M) [45] before and after the gameplay (1920 × 1080 pixels, 1280 × 720 pixels for certain data, 30 fps for each). After playing the game, the participants were asked to watch a recorded video of themselves playing the game and the game screen. While viewing the footage, they identified the time segments (time within the video footage) in which emotions occurred, specified the type of emotion, and rated its intensity (emotional arousal intervals questionnaire). The type of emotion was reported based on the participants’ candid impressions. Intensity was rated on a three-point scale (strong, medium, weak). First, the participants classified each expressed emotion as either “strong” or “weak”; emotions that did not fall into either category were classified as “medium.” An example of the emotional arousal intervals (EAI) questionnaire is listed in Table 2. To avoid playing for a long period and eliminate the influence of the previous gameplay, break and rest times of 5 min each were set after each gameplay. The experiment was conducted using general fluorescent lighting and lighting equipment (NEP Inc., Tokyo, Japan, VARIABLE-29-SET) [46]. The illumination during the experiment was as follows:

- Above the subject: 1260–1750 lx.
- Front of the subject: 640–960 lx.

**Table 2:** Example record in the emotional arousal intervals (EAI) questionnaire.

EAI	Types of Emotions	Intensity	Memo
1:30~1:40	Surprise	Strong	A car suddenly appeared from behind.

This study applied COVID-19 countermeasures, and data were obtained with the consent of the participants in accordance with the ethical regulations on research involving human participants at Akita University.

## 3. Proposed Method

**Figure 3:**
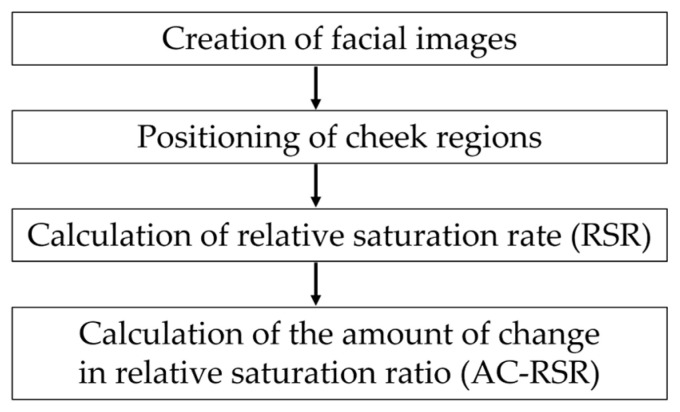
Procedure of the proposed method.

In this study, we focused on how the saturation of the cheek region changed compared with the saturation of the entire face calculated via the L*a*b* color system [47]. The cheek region was selected as the target for analysis because it is one of the facial areas with a high density of blood vessels, and it is easier to capture than the forehead, which may be obscured by hair depending on the participant, or the nose, which is prone to shadows owing to its contours. The procedure for the proposed method is illustrated in Figure 3. First, the captured video image is divided into still images, and a facial image is created by detecting the position of the face in the resulting image. The position of the cheek region is subsequently determined from the facial image. An indicator is calculated through comparisons of the relative saturation of the set cheek region with that of the entire facial region (RSR). Finally, the change in the relative saturation ratio (AC-RSR) is calculated.

### 3.1. Creation of Facial Images

The still images obtained from the filmed video data contained elements that could be disturbances, such as the participant’s clothing and the steering wheel controller of the game. In addition, the face size changed slightly from image to image owing to the variation in the distance between the participant and the camera. To reduce this effect, each still image was normalized to create a facial image.

First, the video images were divided into still images at 30 fps. Thereafter, all still images were targeted, and the positional information of the face, such as the eyes, nose, mouth, and contours, was acquired using the open-source library InsightFace [48]. Finally, the facial regions were extracted based on the acquired positional information and resized. The resizing process uses a single front-facing image captured before the game begins as a reference and resizes the other images. This ensures that the size of each facial image in the videos is the same.

### 3.2. Positioning of Cheek Regions

**Figure 4:**
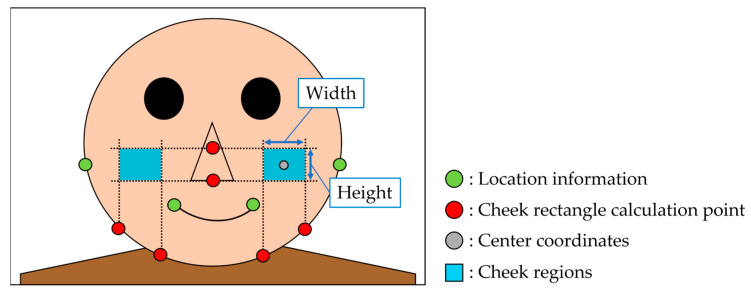
Extraction of facial position information.

The positions of the cheek regions were determined based on the position information of the nose and contours obtained in Section 3.1. The results of facial position information extraction are shown in Figure 4. First, the width of the cheek region was calculated based on the points on the contour shown in red in Figure 4. The height of the cheek region was subsequently calculated from the points on the nose, as shown in red in Figure 4. Consequently, a rectangle was generated from the calculated height and width, and its center coordinates were calculated as a temporary cheek region. This process was performed on all the facial images. The average heights and widths of the temporary cheek area for the entire experimental dataset (three races) were subsequently calculated. The calculated values were used to determine the height and width of the cheek region in each experiment. Finally, the cheek region positions were determined from all the facial images via the determined cheek area sizes and center coordinates. An image of the cheek region is shown in Figure 4. The position of the cheek area was adjusted to ensure that shadows caused by lines did not affect the cheek region.

### 3.3. Calculation of RSR

In this study, we calculated the percentile value [49] of saturation in the cheek region, defined as the RSR, and used it for the analysis.

**Figure 5:**
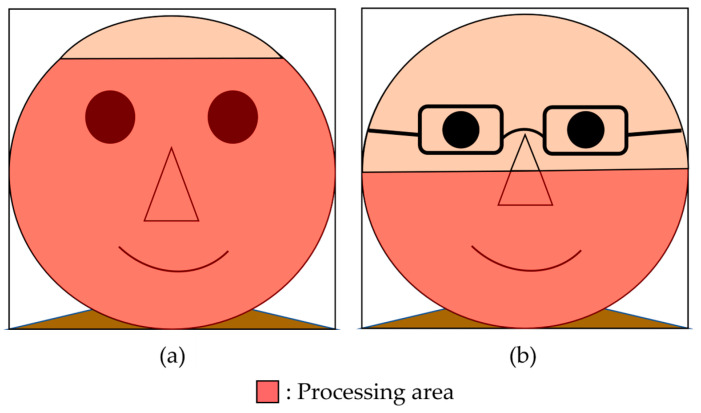
Example of processing area specification. (**a**) Without glasses. (**b**) With glasses.

**Figure 6:**
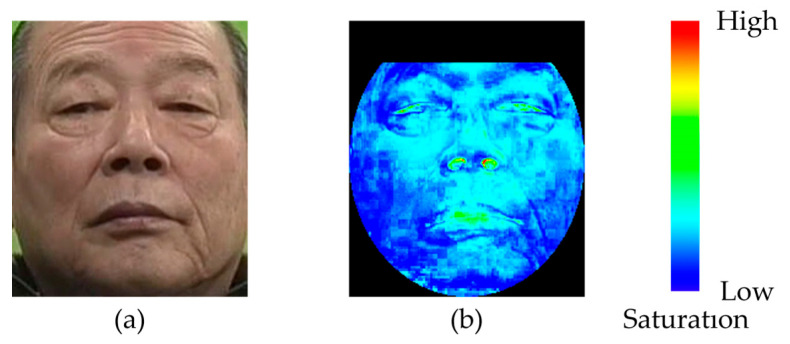
Example of an image after saturation extraction. (**a**) Original image. (**b**) Saturation image.

**Figure 7:**
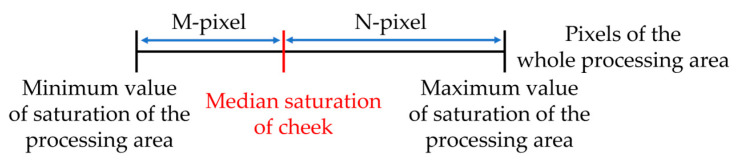
RSR calculation.

**Figure 8:**
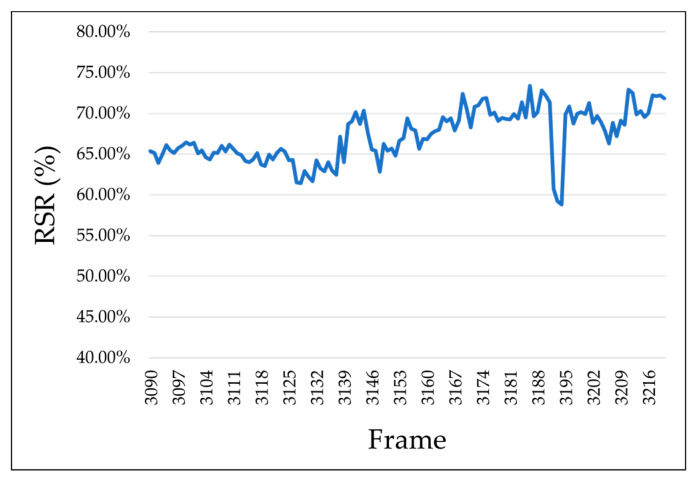
RSR calculation results (Participant A, left cheek).

First, we specified the region where the saturation was extracted (processing region) to remove the background region contained in the facial image. Figure 5 shows an example of how the processing area was specified. Furthermore, an example of a participant’s image after performing the saturation extraction process is presented in Figure 6. If a participant wore eyeglasses, this could affect the saturation. Thus, for participants wearing eyeglasses, as shown in Figure 5b, the area around the glasses was adjusted such that it was excluded from the processing area. Next, the saturation of each pixel in the entire processing area of the facial image was sorted in ascending order. Furthermore, as shown in Figure 7, the numbers of pixels M and N were calculated when the “pixels of the entire processing area” were divided into two parts based on the “median saturation of the cheek area.” The RSR in the cheek area was calculated (for the left and right cheeks) using Equation (1). This process was applied to all facial images to calculate the RSR during gameplay. Figure 8 shows an example of the calculated RSR. The vertical axis in Figure 8 represents the RSR, whereas the horizontal axis indicates the corresponding video frames, which are converted from time (e.g., 1800 frames = 60 s = 1 min). In Equation (1), M and N indicate the number of pixels below and above the median, respectively.

(1) $$\mathrm{RSR}=\frac{M}{M+\;N}$$

### 3.4. Calculation of AC-RSR

**Figure 9:**
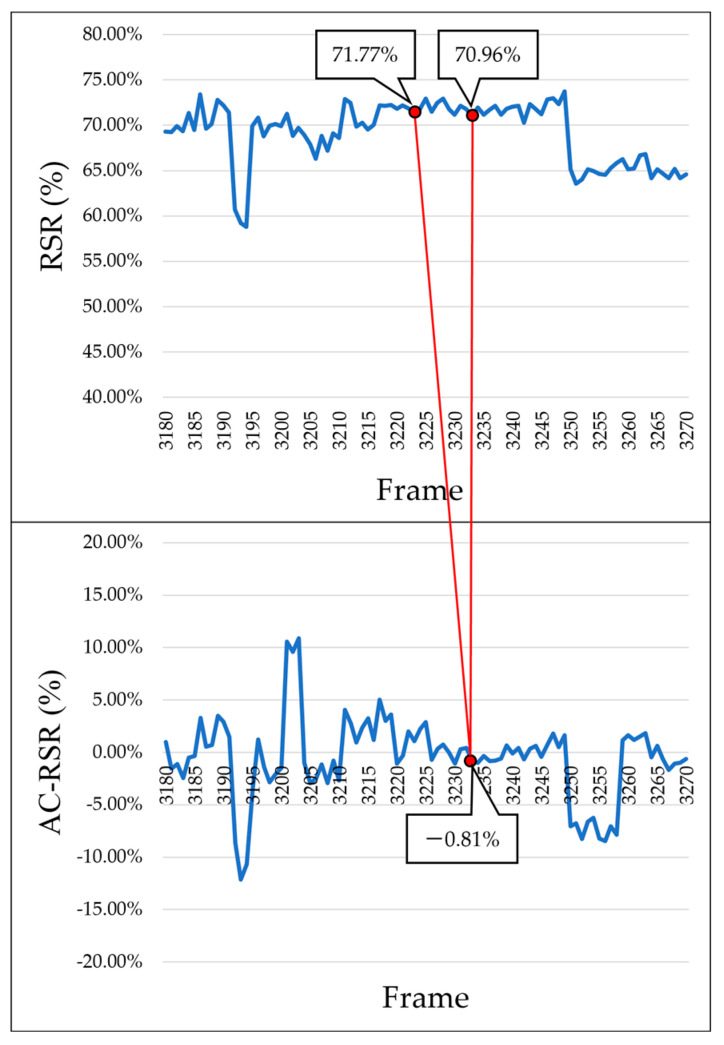
AC-RSR calculation result (Participant A, left cheek).

The AC-RSR was calculated to clarify the change in the calculated RSR time and the direction (increase or decrease) of change. Figure 9 shows an example of the calculated results. In Figure 9, the vertical axes represent the RSR and the AC-RSR, respectively, whereas the horizontal axis indicates the video frames, as in Figure 7. First, the frame of interest (FOI) was determined from the facial images in the video. The difference between the RSR of the FOI frame and the nine frames before the FOI frame was subsequently calculated as the amount of change. The choice of a 9-frame interval is based on a previous study [50], which suggests that there is an approximately 0.2 s time lag between the occurrence of an emotion and the point at which changes appear in the information obtained from the facial surface.

## 4. Analysis Results and Discussion

We analyzed the characteristic changes in facial color during gameplay when emotions were aroused. The RSR calculated in Section 3 and the AC-RSR were used in the analysis. The following two sections were selected for analysis:

- The interval during which “emotional arousal” was evaluated by the participants in the postgame questionnaire (evaluation interval).
- The interval wherein no evaluation was obtained but an event clearly occurred in the gameplay video, such as “passing the other car” or “going out of course” (factor interval).

Both the evaluation and factor intervals were defined using video frame units.

A total of 359 and 455 evaluation and factor intervals, respectively, were analyzed from all the participants. In addition, whether the RSR fluctuated with the occurrence of emotion was determined according to the following two conditions:

- The average AC-RSR in the increasing and decreasing directions during the 10 s before gameplay was calculated. If the calculated average value was used as the threshold value, the amount of change exceeding the threshold value was observed in the evaluation or factor interval. The 10 s before gameplay refers to the time between the start of recording and the beginning of the game, during which the participant was instructed to remain still and look at the camera, serving as baseline data under normal conditions.
- When no change exceeding the threshold was observed, the RSR increased or decreased over time within the analyzed interval.

### 4.1. Discussion Focusing on the Presence or Absence of Fluctuations in the RSR

The RSRs of all analyzed intervals (814 cases) were examined to determine whether a change in RSR occurred during the event time. Fluctuations were defined as an increase, decrease, or increase/decrease in the RSR during the event. The results of the survey indicated that the RSR fluctuated in more than 90% of the intervals: 93% for the left cheek and 94% for the right cheek in the evaluation interval. Moreover, in the factor interval, the RSR varied between 88% and 86% for the left and right cheeks, respectively, and was close to 90%. 

**Figure 10:**
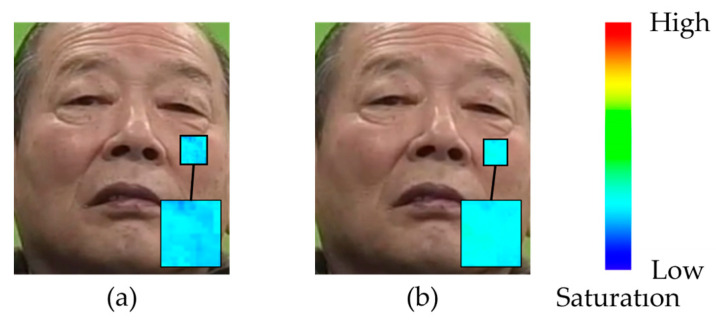
AC-RSR calculation result (Participant A, left cheek). (**a**) Before event. (**b**) After event.

Previous studies [42] have demonstrated that changes in blood flow in facial skin occur in association with emotional responses (e.g., a decrease in cheek temperature caused by blood flow changes was observed as a result of being surprised [51]). Accordingly, the fluctuations in RSR observed in the present study indicate an increase or decrease in facial redness in the cheek region due to changes in blood flow. The fact that such fluctuations were detected within the analysis intervals suggests that the RSR may serve as a potential feature for determining whether an emotion has been elicited in a participant. Figure 10 presents the saturation images of the cheek region before and after an event that triggered an emotional response. In this example, the images were captured before and after overtaking multiple other vehicles, and the participant self-reported the emotion of “joy” after overtaking. A comparison of the images before and after the event reveals an overall increase in cheek saturation following the event, implying that blood flow changes associated with emotion elicitation occurred.

### 4.2. Discussion Focusing on the Fluctuation in RSR in the Evaluation Interval

**Figure 11:**
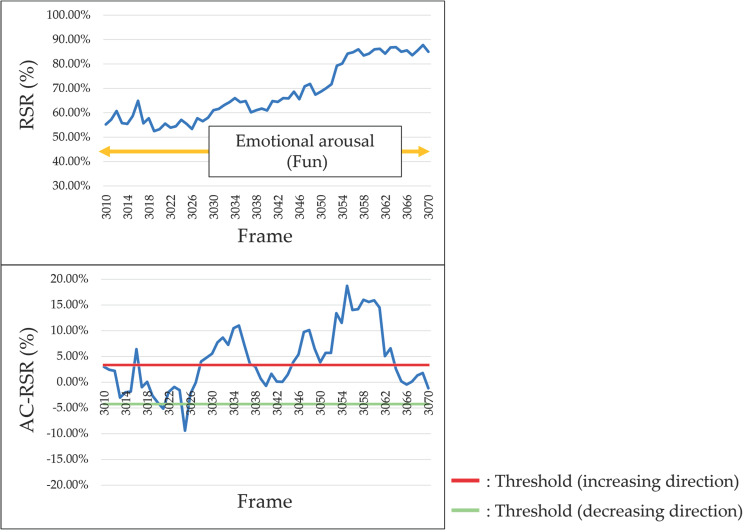
Example of RSR and AC-RSR calculation results during positive emotions (Participant C, left cheek).

**Figure 12:**
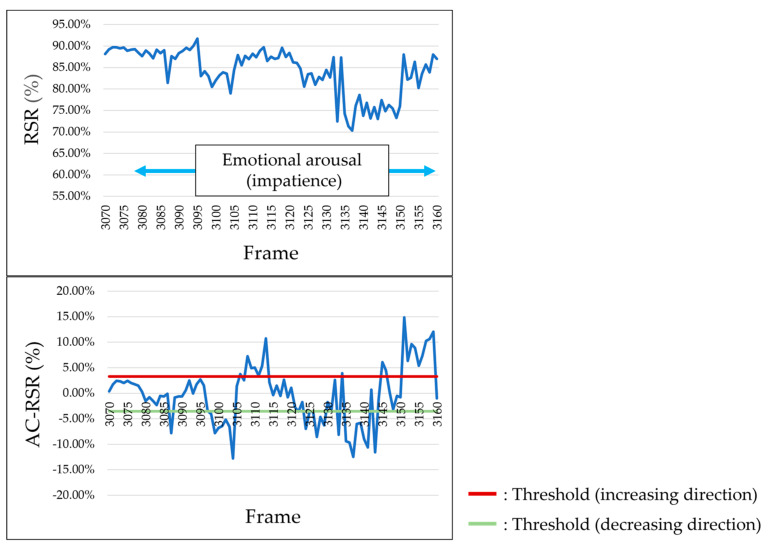
Example of RSR and AC-RSR calculation results during negative emotions (Participant M, left cheek).

According to the results of the survey reported in Section 4.1, the RSR increased (i.e., facial redness increased) when participants experienced positive emotions such as joy and happiness. Figure 11 shows the results for participants who exhibited an increase in the RSR during the occurrence of positive emotions. However, when negative emotions such as “surprise”, “impatience”, or “frustration” occurred, the RSR decreased (i.e., facial redness decreased). Figure 12 shows the results for participants who showed a decrease in the RSR during the occurrence of negative emotions.

On the basis of the above results, we hypothesized that the change in the RSR may be characteristic of the type of emotion that occurred in the participant and conducted a survey during the evaluation interval. Specifically, the following characteristics were investigated to determine whether they were observed in the actual fluctuations:

- RSR increases when positive emotions occur.
- RSR decreases when negative emotions occur.

The results of these investigations are summarized in Table 3. In Table 3, the first column indicates the participant, the second column denotes which experimental session (first or second) the data were collected from, the third column shows the total number of evaluation intervals across three gameplay sessions, and the fourth and fifth columns show the number of cases in which the hypothesized pattern was observed in the respective cheek region. Among the total evaluation intervals, 68% were for the left cheek and 70% were for the right cheek, indicating the expected fluctuations in the interval close to 70%. This result indicates that the RSR tended to increase and decrease with positive and negative emotions, respectively.

**Figure 13:**
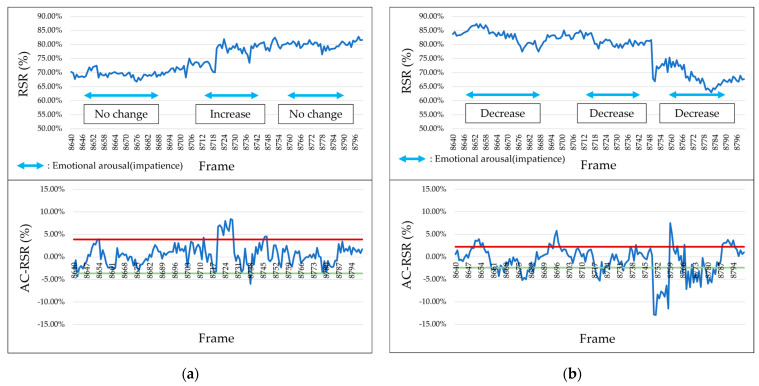
RSR and AC-RSR calculation results (Participant F). (**a**) Left cheek. (**b**) Right cheek.

In addition, Table 3 lists that certain participants represented less than 50% of the interval in which the expected fluctuations were observed. However, when focusing on the other cheek regions of the participants, the expected fluctuation was observed in all participants in more than 50% of the intervals. The RSR values calculated for these intervals are shown in Figure 13. In certain cases, the RSR fluctuated only in the left or right cheeks; in others, the RSR fluctuated in the opposite direction on the left and right cheeks. Therefore, rather than analyzing changes in the RSR of the left and right cheeks separately, it is considered more important to focus on the relationship between the changes observed in both cheeks during emotional arousal. Moreover, this asymmetry in fluctuations between the left and right cheeks has potential for use as a feature for estimating the type of emotion elicited in a participant. Therefore, further investigation focusing on the relationship between the left and right cheek regions is planned for future work.

To conduct a more detailed analysis, we investigated the extent to which the expected fluctuations were observed in the evaluation intervals of each video for each participant. Specifically, for each video, we calculated the agreement rate for the left and right cheeks separately—defined as the proportion of evaluation intervals in which the expected fluctuations were observed. As a result, 29 out of 105 videos showed agreement rates below 80% on both cheeks. Focusing on the questionnaire results completed by the participants, we observed that approximately 70% (21 out of 29) of these videos were either of participants who reported feeling nervous or anxious before the experiment or gameplay, or from the first gameplay session of participants who were participating in the experiment for the first time. Among the remaining eight videos, one corresponded to a participant who reported in the questionnaire that they had already felt fatigued before the experiment. This suggests that when participants are inexperienced or feel nervous and anxious because of a new experience, or when they are fatigued, the expected trends may not always be observed due to their psychological or physical condition being different from usual. Additionally, in the remaining seven videos, the participants did not report feeling nervous or anxious. In this study, evaluation intervals were set based on the participants’ subjective evaluations. Therefore, there may be instances where the intervals reported in the questionnaire do not necessarily correspond to the actual periods when emotions were experienced. We believe that the remaining seven videos might be influenced by this discrepancy.

### 4.3. Discussion Focusing on the Fluctuation in the RSR in the Factor Interval

We investigated whether the characteristics observed in Section 4.2 were also observed in the factor interval. Specifically, the following characteristics were investigated to determine whether they were observed in the actual fluctuations:

- The RSR increases when an event may induce positive emotions, such as “passing the other car” or “obtaining the first position.”
- The RSR decreases when an event might induce negative emotions, such as “going off course” or “crashing into a wall.”

The results of these investigations are summarized in Table 4. In Table 4, the first column indicates the participant, the second column denotes which experimental session (first or second) the data were collected from, the third column shows the total number of factor intervals across three gameplay sessions, and the fourth and fifth columns show the number of cases in which the hypothesized pattern was observed in the respective cheek region. Among all the factor intervals, 57% and 55% of the intervals for the left and right cheeks, respectively, exhibited the expected fluctuations. Although this percentage was lower than that of the evaluation intervals, the expected fluctuation was observed in approximately 60% of the intervals, indicating that the RSR showed characteristic movements depending on the type of emotion. The factor interval was defined as the interval in which the participants did not evaluate the occurrence of emotions. Therefore, focusing on changes in the RSR indicates the possibility of estimating the occurrence of emotions that participants themselves were unaware of. However, the occurrence or absence of an emotion in this study is based on the participants’ subjective evaluations. Hence, we will combine different evaluation metrics in future research for further examination. 

### 4.4. Consideration Based on Participants’ Experience

The participants’ prior experience with video games may have influenced the experimental results. Therefore, cases in which participants’ experience likely affected the occurrence of emotions during gameplay are discussed in this section.

Table 5 summarizes the number of positive and negative emotions reported during each of the three gameplay sessions in Experimental Session 1 and Experimental Session 2, respectively. As listed in Table 5, the number of positive emotions was lowest during the first gameplay in Session 1 and increased as the participants played more games. Compared with that in Session 1, the number of positive emotions in Session 2 increased across all three gameplay trials. Conversely, the number of negative emotions was notably greater than that of positive emotions starting from the first gameplay in Session 1. Although this trend continued in the second and third gameplay trials in Session 1, the number of negative emotions decreased in Session 2 compared with Session 1.

Such emotional arousal may have been influenced by the participants’ experiences. (1) None of the participants had played the racing game used in this experiment prior to the study. Consequently, 7 out of 19 participants reported feeling nervous or confused during the initial gameplay because they had never played the game used in the experiment. (2) As noted in Section 2, all the participants had experience driving real vehicles. Due to this, 15 out of 19 participants reported feeling confused by the differences in handling and control (e.g., steering response and acceleration sensitivity) between the game and real-world driving. All participants who experienced such confusion due to factors (1) and (2) reported that these feelings diminished as they continued to play, although the rate at which this occurred varied among individuals.

Therefore, it can be inferred that, in the early stages of the experiment, the participants’ prior experiences described in (1) and (2) may have contributed to the frequent occurrence of negative emotions. As participants gained experience through repeated gameplay, these influences may have lessened, resulting in fewer negative emotions and more opportunities for the emergence of positive emotions. These findings suggest that, in the context of this study, which aims to clarify the potential benefits of eSports for promoting healthy longevity, repeated gameplay experience is important for enabling older adults to enjoy eSports.

However, the influence of factor (2) can be regarded as a characteristic of the present experiment, in which participants with extensive real-world driving experience played a racing game. Furthermore, the fact that the influences of both (1) and (2) were resolved relatively early through repeated gameplay may also have been facilitated by the ease of operation of the game and the participants’ driving experience. Therefore, when a different game is used, it is possible that more time will be required for nervousness or anxiety toward the game to subside, and this should be taken into consideration when designing the experiment.

### 4.5. Comparison with Facial Expression Recognition Technology

In this experiment, it was confirmed that characteristic fluctuations in the RSR occur during emotion elicitation. Finally, to evaluate the effectiveness of the proposed method, a comparison with existing techniques was conducted.

Although various emotion estimation methods exist, in this study we compare the proposed method with emotion estimation techniques based on facial expressions. Specifically, among the emotion classification models included in the Py-Feat package provided in [52], we employed the Residual Masking Network (RMN) model [53], which achieved the highest classification accuracy in [52]. The RMN model is a CNN-based deep learning model that classifies an input facial image into one of seven emotions (anger, disgust, fear, happiness, sadness, surprise, and neutral). Unlike the RMN model, the proposed method does not classify emotions; instead, it detects changes in facial saturation information associated with emotion elicitation. Therefore, in this comparative experiment, both the proposed method and the RMN model were applied to the evaluation intervals to examine whether (1) fluctuations in the RSR were observed and (2) the emotion was judged to be elicited (i.e., the output was other than “neutral”).

**Figure 14:**
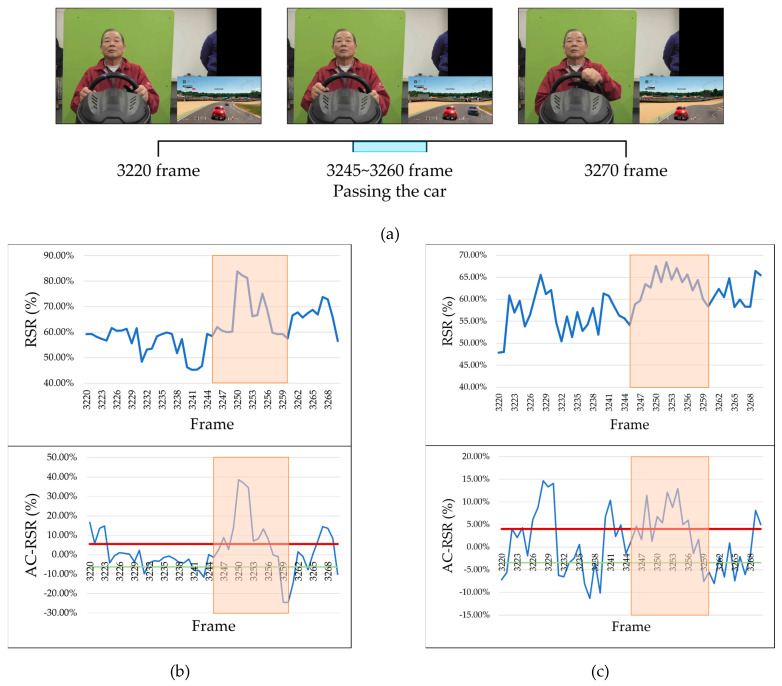
Results of face image, RSR, and AC-RSR calculations in the EAI (Participant A). (**a**) Face images in the EAI. (**b**) Left cheek. (**c**) Right cheek.

Figure 14 presents an example of facial images from Participant A during an emotion-eliciting interval, along with the calculated RSR and AC-RSR for the left and right cheek regions. This interval corresponds to a moment in which the participant successfully overtook a competitor’s car and self-reported the emotion of “joy.” As shown in Figure 14b,c, an increase in the RSR at the time of overtaking was detected using the proposed method. As discussed in Section 4.2, RSR tends to increase during the elicitation of positive emotions; therefore, the observed saturation change in this interval is consistent with the participant’s self-reported emotion of “joy.” In contrast, the RMN model’s output for the same interval classified all 51 frames, from frame 3220 to frame 3270, as “neutral.” As can be seen from the sample images in Figure 14a, the participant’s facial expression showed minimal change immediately before and after overtaking the competitor’s car. This likely explains why the RMN model output was “neutral.”

As noted in Section 1, people can voluntarily control their facial expressions and may choose not to express their actual emotional state through facial appearance. Therefore, these comparative results indicate that the proposed method has the potential to estimate the emotions of participants whose emotions are difficult to infer from facial expressions alone. This advantage arises from the fact that the proposed method focuses on physiological changes that cannot be voluntarily controlled.

## 5. Conclusions

In this study, we propose a method for analyzing facial color and the characteristics of changes in facial color during emotional arousal in Japanese older adult participants while playing eSports. The results of this study are summarized as follows:

(1) The fluctuation in the RSR can be used as a feature to judge whether an emotion occurs in a participant.
(2) The RSR tended to increase with positive emotions and decrease with negative emotions.
(3) Focusing on the relationship between the fluctuations in the RSRs of both cheeks has the potential to be used as a feature to estimate the type of emotion that is elicited in a participant.
(4) Focusing on the change in the RSR indicated a possibility of estimating the occurrence of emotions that participants themselves are unaware of.
(5) The proposed method, which focuses on physiological changes in facial saturation, demonstrated the potential to estimate the emotions of participants whose emotions are difficult to infer from facial expressions alone.

The analysis method proposed in this study focuses on facial color, a physiological change that is measurable with a noncontact method and difficult for individuals to consciously control. Distinctive changes in facial color were observed during emotional arousal. This finding is expected to contribute to the advancement of emotion recognition technologies. Furthermore, data collection targeting older adults engaged in eSports, as well as studies on their physiological and psychological responses during gameplay, remain limited. Therefore, this research is also expected to contribute to the further development of this field. Additionally, by exploring the use of emotional information to assess the psychological state of older adults participating in eSports, this study opens new possibilities. It may aid in evaluating their engagement in eSports activities as a means to promote healthy longevity.

The facial color characteristics observed during emotional arousal in this study may be useful for developing technologies that estimate emotions during eSports activities. In addition, they have potential applications in a variety of fields. For example, if it becomes possible to estimate a person’s emotions from changes in facial color and share the results with others, this could be applied to technologies that support communication for individuals who have difficulty expressing their emotions outwardly, as well as to systems for remote communication assistance that help interlocutors understand each other’s emotions during conversations over long distances. Furthermore, if patterns of emotional arousal—such as a tendency to experience positive emotions—can be recorded for each individual, and deviations from these patterns can be detected and notified to others, this could contribute to the development of technologies for monitoring an individual’s physical or mental condition from a remote location.

However, there are several limitations in the present study. The assessment of emotional arousal during gameplay relied on participants’ subjective reports, which means that there may be discrepancies between the actual timing of an emotion occur-ring and the reported timing. Future studies should incorporate multiple evaluation indicators, such as the use of physiological sensors (e.g., heart rate monitors). In addition, the questionnaire format used for evaluating elicited emotions should also be re-considered. Moreover, the facial color changes examined in this study occur with the emergence of emotions, but they may also be triggered by other physiological states, such as changes in health conditions or fatigue. As noted in Section 4.2, in this experiment, some participants who reported feeling nervous or fatigued before the session did not always exhibit the expected facial color fluctuations. Therefore, future work should also consider multimodal approaches that combine facial color changes with other features, such as eye gaze, blinking, or physiological signals like heart rate. We believe that such multimodal approaches will also be essential for future investigations that aim to capture more complex information, such as the type and intensity of emotions experienced by participants. This study included 19 participants. To examine whether the changes in facial color observed during emotional arousal are generalizable, future research should involve a larger number of older adult participants. Furthermore, in order to enable broader applications of this technology beyond eSports, experiments should be conducted using facial image data from different age groups, from people in different cultural backgrounds, and with varying skin tones, to determine whether the facial color changes identified in this study are universally applicable. In addition, only 2 of the 19 participants regularly played video games, making it difficult to conduct sub-group analyses comparing participants with extensive gaming experience to those without. Therefore, further experiments should be conducted, and the discussion should be revisited with a sample comprising a sufficient number of participants with gaming experience.

Finally, for future directions, building on the distinctive changes observed in cheek color during emotional arousal, we plan to develop a method for estimating when emotions arise in older adults during eSports activities. Furthermore, we plan to investigate methods for estimating not only the timing but also the type and intensity of emotions by focusing on combinations of facial color with other features. In addition, in this experiment, data were collected without major changes in lighting conditions. We plan to examine how variations in the recording environment, such as changes in illumination, affect the observation of facial color fluctuations associated with emotional arousal. 

## Figures and Tables

**Table 1:** Results of the participant survey.

	Participants with Driving Experience (Proportion)	Participants with Gaming Experience (Proportion)
Have experienced at least once	19 (100%)	10 (52%)
Engage regularly	18 (94%)	2 (20%)

**Table 3:** RSR survey results in the evaluation intervals.

Participant	Experimental Session	Number of Evaluation Intervals	Fluctuations Observed	
			Left Cheek	Right Cheek
A	1	7	6 (86%)	6 (86%)
	2	18	9 (50%)	11 (61%)
B	1	18	12 (67%)	13 (72%)
	2	7	5 (71%)	4 (57%)
C	1	11	8 (73%)	10 (91%)
	2	14	10 (71%)	10 (71%)
D	1			
	2	7	5 (71%)	5 (71%)
E	1	16	11 (69%)	10 (63%)
	2	16	12 (75%)	7 (44%)
F	1	22	11 (50%)	15 (68%)
	2	13	5 (38%)	10 (77%)
G	1	7	6 (86%)	7 (100%)
	2	10	7 (70%)	10 (100%)
H	1	11	9 (82%)	10 (91%)
	2	9	8 (89%)	9 (100%)
I	1	7	5 (71%)	7 (100%)
	2	7	7 (100%)	6 (86%)
J	1	20	14 (70%)	10 (50%)
	2	8	6 (75%)	6 (75%)
K	1	7	6 (86%)	4 (57%)
	2	9	5 (56%)	4 (44%)
L	1	17	13 (76%)	10 (59%)
	2	13	8 (62%)	9 (69%)
M	1	11	6 (55%)	6 (55%)
	2	8	5 (63%)	6 (75%)
N	1	13	6 (46%)	8 (62%)
	2	4	1 (25%)	3 (75%)
O	1	20	15 (75%)	14 (70%)
	2			
P	1	11	9 (82%)	7 (64%)
	2	2	1 (50%)	2 (100%)
Q	1	0	0	0
	2	0	0	0
R	1	6	5 (83%)	4 (67%)
	2	2	2 (100%)	2 (100%)
S	1			
	2	8	6 (75%)	5 (63%)

Red text: cases below 50%.

**Table 4:** RSR survey results in the factor intervals.

Participant	Experimental Session	Number of Factor Intervals	Fluctuations Observed	
			Left Cheek	Right Cheek
A	1	11	5 (45%)	7 (64%)
	2	3	3 (100%)	2 (67%)
B	1	14	5 (36%)	7 (50%)
	2	26	20 (77%)	12 (46%)
C	1	14	11 (79%)	9 (64%)
	2	2	1 (50%)	2 (100%)
D	1			
	2	8	5 (63%)	4 (50%)
E	1	9	5 (56%)	6 (67%)
	2	9	5 (56%)	3 (33%)
F	1	11	5 (45%)	4 (36%)
	2	9	7 (78%)	4 (44%)
G	1	4	2 (50%)	3 (75%)
	2	7	4 (57%)	5 (71%)
H	1	4	3 (75%)	4 (100%)
	2	12	7 (58%)	7 (58%)
I	1	9	6 (67%)	7 (78%)
	2	8	4 (50%)	3 (38%)
J	1	13	8 (62%)	6 (46%)
	2	18	5 (28%)	10 (56%)
K	1	12	6 (50%)	8 (67%)
	2	8	5 (63%)	4 (50%)
L	1	14	7 (50%)	8 (57%)
	2	27	19 (70%)	12 (44%)
M	1	10	7 (70%)	5 (50%)
	2	17	11 (65%)	8 (47%)
N	1	15	8 (53%)	11 (73%)
	2	13	9 (69%)	7 (54%)
O	1	6	4 (67%)	2 (33%)
	2			
P	1	4	3 (75%)	2 (50%)
	2	20	8 (40%)	9 (45%)
Q	1	38	20 (53%)	21 (55%)
	2	12	6 (50%)	4 (33%)
R	1	12	4 (33%)	4 (33%)
	2	13	6 (46%)	8 (62%)
S	1			
	2	43	25 (58%)	30 (70%)

Red text: cases below 50%.

**Table 5:** Number of occurrences of positive and negative emotions.

		Number of Emotion Occurrences	
		Experimental Session 1	Experimental Session 2
Positive emotion	play1	9	11
	play2	19	22
	play3	14	18
Negative emotion	play1	36	23
	play2	81	45
	play3	45	36

## Data Availability

The data presented in this study are available on request from the corresponding author due to privacy concerns regarding the study participants.

## Abbreviations

The following abbreviations are used in this manuscript:

eSports	Electronic Sports
EAI	Emotional Arousal Intervals
RSR	Relative Saturation Rate
AC-RSR	Amount of Change in Relative Saturation Rate
FOI	Frame of Interest
